# (*E*)-Methyl 1,3-thia­zol-2-yl ketone 2,4-dinitro­phenyl­hydrazone

**DOI:** 10.1107/S1600536808015298

**Published:** 2008-05-24

**Authors:** Shang Shan, Yu-Liang Tian, Shan-Heng Wang, Wen-Long Wang, Ying-Li Xu

**Affiliations:** a College of Chemical Engineering and Materials Science, Zhejiang University of Technology, People’s Republic of China

## Abstract

Crystals of the title compound, C_11_H_9_N_5_O_4_S, were obtained from a condensation reaction of 2,4-dinitro­phenyl­hydrazine and methyl 1,3-thia­zol-2-yl ketone. Excluding two methyl H atoms, the mol­ecule displays a planar structure, the dihedral angle between the terminal thia­zole and benzene rings being 1.82 (8)°. The imino group links with adjacent nitro and thia­zole groups by intra­molecular bifurcated hydrogen bonding. The centroid–centroid separation of 3.7273 (11) Å between nearly parallel benzene and thia­zole rings of adjacent mol­ecules indicates the existence of π–π stacking in the crystal structure. Weak inter­molecular C—H⋯O hydrogen bonding is also observed.

## Related literature

For general background, see: Okabe *et al.* (1993 ▶); Shan *et al.* (2003 ▶, 2006 ▶). For a related structure, see: Shan *et al.* (2008 ▶).
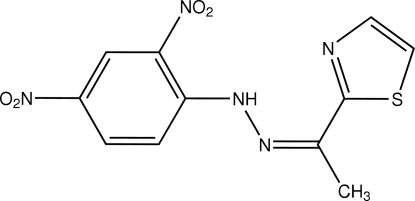


## Experimental

### 

#### Crystal data

C_11_H_9_N_5_O_4_S
*M* *_r_* = 307.29
Monoclinic, 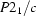

*a* = 8.0126 (5) Å
*b* = 7.3239 (4) Å
*c* = 21.8683 (12) Å
β = 92.610 (2)°
*V* = 1281.98 (13) Å^3^
*Z* = 4
Mo *K*α radiation
μ = 0.28 mm^−1^
*T* = 295 (2) K
0.46 × 0.42 × 0.36 mm

#### Data collection

Rigaku R-AXIS RAPID IP diffractometer
Absorption correction: none
12253 measured reflections
2943 independent reflections
1845 reflections with *I* > 2σ(*I*)
*R*_int_ = 0.039

#### Refinement

*R*[*F* ^2^ > 2σ(*F* ^2^)] = 0.038
*wR*(*F* ^2^) = 0.119
*S* = 1.11
2943 reflections
192 parameters
H-atom parameters constrained
Δρ_max_ = 0.22 e Å^−3^
Δρ_min_ = −0.19 e Å^−3^

### 

Data collection: *PROCESS-AUTO* (Rigaku, 1998 ▶); cell refinement: *PROCESS-AUTO*; data reduction: *CrystalStructure* (Rigaku/MSC, 2002 ▶); program(s) used to solve structure: *SIR92* (Altomare *et al.*, 1993 ▶); program(s) used to refine structure: *SHELXL97* (Sheldrick, 2008 ▶); molecular graphics: *ORTEP-3 for Windows* (Farrugia, 1997 ▶); software used to prepare material for publication: *WinGX* (Farrugia, 1999 ▶).

## Supplementary Material

Crystal structure: contains datablocks I, global. DOI: 10.1107/S1600536808015298/om2236sup1.cif

Structure factors: contains datablocks I. DOI: 10.1107/S1600536808015298/om2236Isup2.hkl

Additional supplementary materials:  crystallographic information; 3D view; checkCIF report

## Figures and Tables

**Table 1:** Hydrogen-bond geometry (Å, °)

*D*—H⋯*A*	*D*—H	H⋯*A*	*D*⋯*A*	*D*—H⋯*A*
N3—H3⋯O1	0.86	2.03	2.628 (2)	126
N3—H3⋯N5	0.86	2.03	2.686 (2)	133
C9—H9⋯O1^i^	0.93	2.58	3.289 (3)	133

Symmetry code: (i) 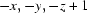.

**Fig. 1.**
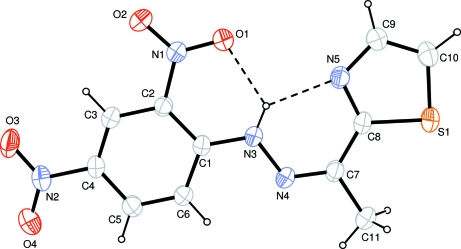
The molecular structure of the title compound with 30% probability displacement ellipsoids for non-H atoms, dashed lines indicate hydrogen bonding.

**Fig. 2.**
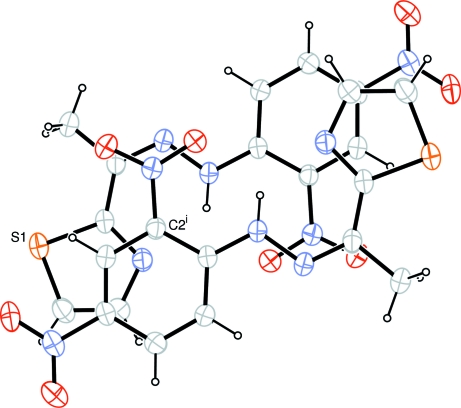
A diagram showing the partially overlapped arrangement of benzene and thiazole rings [symmetry code: (i) 1 - x,1 - y,1 - z].

## supplementary crystallographic information

### Comment

As some phenylhydrazone derivatives have shown to be potentially DNA damaging and mutagenic agents (Okabe *et al.*, 1993), a series of new phenylhydrazone derivatives have been synthesized in our laboratory (Shan *et al.*, 2003; Shan *et al.*, 2006). As part of the ongoing investigation, the title compound has recently been prepared and its crystal structure is reported here.

The molecular structure of the title compound is shown in Fig. 1. The molecule displays a coplanar structure, except methyl H atoms, the dihedral angle between the thiazole and benzene rings being 1.82 (8)°. The N4—C7 bond distance is significantly shorter than N3—N4 and N3—C1 bond distances (Table 1), and indicates the typical C?N double bond. The phenylhydrazone and thiazole are located on the opposite sides of the C?N double bond, the molecule has an E-configuration, similar to that found in a related compound, (E)-2-furlyl methylketone 2,4-dinitrophenylhydrazone (Shan *et al.*, 2008). The imino group links with adjacent nitro and thiazole groups by intra-molecular bifurcated hydrogen bonding (Fig. 1 and Table 2).

A partially overlapped arrangement between nearly parallel benzene ring and thiazole ring of the adjacent molecule is illustrated in Fig. 2. The dihedral angle and centroid-to-centroid separation are 1.82 (8)° and 3.7273 (11) Å, these suggest the existence of π-π stacking between adjacent molecules in the crystal. The crystal structure also contains intermolecular weak C—H···O hydrogen bonding (Table 2).

### Experimental

2,4-Dinitrophenylhydrazine (0.4 g, 2 mmol) was dissolved in ethanol (10 ml), and H_2_SO_4_ solution (98%, 0.5 ml) was slowly added to the ethanol solution with stirring. The solution was heated at 333 K for several min until the solution cleared. 2-Thiazolyl methyl ketone (0.25 g, 2 mmol) was added to the above solution with continuous stirring, and the mixture was refluxed for 30 min. When the solution had cooled to room temperature yellow powder crystals appeared. The powder crystals were separated and washed with water three times. Recrystallization from an absolute ethanol yielded well shaped single crystals.

### Refinement

Methyl H atoms were placed in calculated positions with C—H = 0.96 Å and the torsion angle was refined to fit the electron density, *U*_iso_(H) = 1.5*U*_eq_(C). Other H atoms were placed in calculated positions with C—H = 0.93 and N—H = 0.86 Å, and refined in riding mode with *U*_iso_(H) = 1.2*U*_eq_(C,*N*).

### Crystal data

**Table:**

C_11_H_9_N_5_O_4_S	*F*_000_ = 632
*M**_r_* = 307.29	*D*_x_ = 1.592 Mg m^−^^3^
Monoclinic, *P*2_1_/*c*	Mo *K*α radiation λ = 0.71073 Å
Hall symbol: -P 2ybc	Cell parameters from 5366 reflections
*a* = 8.0126 (5) Å	θ = 3.1–25.5º
*b* = 7.3239 (4) Å	µ = 0.28 mm^−^^1^
*c* = 21.8683 (12) Å	*T* = 295 (2) K
β = 92.610 (2)º	Prism, orange
*V* = 1281.98 (13) Å^3^	0.46 × 0.42 × 0.36 mm
*Z* = 4	

### Data collection

**Table:**

Rigaku R-AXIS RAPID IP diffractometer	2943 independent reflections
Radiation source: fine-focus sealed tube	1845 reflections with *I* > 2σ(*I*)
Monochromator: graphite	*R*_int_ = 0.039
Detector resolution: 10.00 pixels mm^-1^	θ_max_ = 27.5º
*T* = 295(2) K	θ_min_ = 3.1º
ω scans	*h* = −10→10
Absorption correction: none	*k* = −9→8
12253 measured reflections	*l* = −28→28

### Refinement

**Table:**

Refinement on *F*^2^	Hydrogen site location: inferred from neighbouring sites
Least-squares matrix: full	H-atom parameters constrained
*R*[*F*^2^ > 2σ(*F*^2^)] = 0.038	*w* = 1/[σ^2^(*F*_o_^2^) + (0.0544*P*)^2^ + 0.1169*P*] where *P* = (*F*_o_^2^ + 2*F*_c_^2^)/3
*wR*(*F*^2^) = 0.119	(Δ/σ)_max_ < 0.001
*S* = 1.11	Δρ_max_ = 0.22 e Å^−^^3^
2943 reflections	Δρ_min_ = −0.19 e Å^−^^3^
192 parameters	Extinction correction: SHELXL97 (Sheldrick, 2008), Fc^*^=kFc[1+0.001xFc^2^λ^3^/sin(2θ)]^-1/4^
Primary atom site location: structure-invariant direct methods	Extinction coefficient: 0.029 (3)
Secondary atom site location: difference Fourier map	

### Special details

**Table:**

Geometry. All e.s.d.'s (except the e.s.d. in the dihedral angle between two l.s. planes) are estimated using the full covariance matrix. The cell e.s.d.'s are taken into account individually in the estimation of e.s.d.'s in distances, angles and torsion angles; correlations between e.s.d.'s in cell parameters are only used when they are defined by crystal symmetry. An approximate (isotropic) treatment of cell e.s.d.'s is used for estimating e.s.d.'s involving l.s. planes.
Refinement. Refinement of *F*^2^ against ALL reflections. The weighted *R*-factor *wR* and goodness of fit *S* are based on *F*^2^, conventional *R*-factors *R* are based on *F*, with *F* set to zero for negative *F*^2^. The threshold expression of *F*^2^ > σ(*F*^2^) is used only for calculating *R*-factors(gt) *etc*. and is not relevant to the choice of reflections for refinement. *R*-factors based on *F*^2^ are statistically about twice as large as those based on *F*, and *R*- factors based on ALL data will be even larger.

### Fractional atomic coordinates and isotropic or equivalent isotropic displacement parameters (Å^2^)

**Table:**

	*x*	*y*	*z*	*U*_iso_*/*U*_eq_	
S1	0.18922 (7)	0.17161 (8)	0.69372 (2)	0.0610 (2)	
N1	0.3851 (2)	0.1710 (2)	0.39844 (7)	0.0550 (4)	
N2	0.9262 (2)	0.3699 (2)	0.33512 (8)	0.0614 (5)	
N3	0.49462 (19)	0.2538 (2)	0.52598 (6)	0.0488 (4)	
H3	0.3946	0.2130	0.5201	0.059*	
N4	0.5583 (2)	0.2973 (2)	0.58348 (7)	0.0523 (4)	
N5	0.20975 (19)	0.1562 (2)	0.57733 (7)	0.0508 (4)	
O1	0.28243 (18)	0.1495 (2)	0.43763 (7)	0.0762 (5)	
O2	0.35547 (19)	0.1322 (2)	0.34454 (7)	0.0777 (5)	
O3	0.8766 (2)	0.3374 (3)	0.28262 (7)	0.0865 (5)	
O4	1.0639 (2)	0.4328 (2)	0.34849 (8)	0.0820 (5)	
C1	0.5967 (2)	0.2783 (2)	0.47889 (8)	0.0436 (4)	
C2	0.5482 (2)	0.2422 (2)	0.41680 (8)	0.0439 (4)	
C3	0.6570 (2)	0.2710 (2)	0.37046 (8)	0.0475 (4)	
H3A	0.6232	0.2463	0.3301	0.057*	
C4	0.8131 (2)	0.3355 (2)	0.38400 (8)	0.0487 (5)	
C5	0.8673 (2)	0.3689 (3)	0.44448 (9)	0.0565 (5)	
H5	0.9754	0.4101	0.4534	0.068*	
C6	0.7610 (2)	0.3408 (3)	0.49027 (8)	0.0531 (5)	
H6	0.7982	0.3638	0.5304	0.064*	
C7	0.4663 (2)	0.2727 (3)	0.62965 (8)	0.0498 (4)	
C8	0.2965 (2)	0.2023 (2)	0.62794 (8)	0.0469 (4)	
C9	0.0559 (3)	0.0960 (3)	0.59096 (9)	0.0571 (5)	
H9	−0.0221	0.0579	0.5608	0.069*	
C10	0.0226 (3)	0.0947 (3)	0.65099 (10)	0.0622 (5)	
H10	−0.0778	0.0568	0.6666	0.075*	
C11	0.5480 (3)	0.3213 (3)	0.69085 (9)	0.0675 (6)	
H11A	0.6545	0.3770	0.6849	0.101*	
H11B	0.5635	0.2127	0.7150	0.101*	
H11C	0.4781	0.4052	0.7116	0.101*	

### Atomic displacement parameters (Å^2^)

**Table:**

	*U*^11^	*U*^22^	*U*^33^	*U*^12^	*U*^13^	*U*^23^
S1	0.0664 (4)	0.0751 (4)	0.0428 (3)	0.0004 (3)	0.0172 (2)	−0.0027 (2)
N1	0.0516 (9)	0.0714 (11)	0.0421 (9)	−0.0039 (8)	0.0013 (7)	0.0003 (8)
N2	0.0643 (11)	0.0655 (11)	0.0558 (11)	−0.0015 (9)	0.0195 (9)	0.0047 (9)
N3	0.0477 (8)	0.0626 (10)	0.0361 (8)	−0.0014 (7)	0.0045 (7)	−0.0016 (7)
N4	0.0549 (10)	0.0648 (10)	0.0371 (8)	−0.0011 (8)	0.0022 (7)	−0.0041 (7)
N5	0.0541 (9)	0.0563 (9)	0.0424 (9)	0.0006 (7)	0.0060 (7)	0.0014 (7)
O1	0.0560 (9)	0.1221 (14)	0.0507 (9)	−0.0217 (8)	0.0066 (7)	−0.0024 (8)
O2	0.0684 (9)	0.1195 (13)	0.0448 (8)	−0.0168 (8)	−0.0027 (7)	−0.0133 (8)
O3	0.0966 (12)	0.1204 (14)	0.0444 (9)	−0.0222 (10)	0.0232 (8)	−0.0015 (9)
O4	0.0623 (10)	0.1066 (13)	0.0786 (11)	−0.0139 (9)	0.0215 (8)	0.0070 (10)
C1	0.0478 (10)	0.0452 (9)	0.0382 (9)	0.0027 (8)	0.0062 (8)	0.0007 (8)
C2	0.0456 (10)	0.0477 (10)	0.0385 (9)	0.0024 (8)	0.0026 (8)	0.0017 (8)
C3	0.0560 (11)	0.0490 (10)	0.0377 (9)	0.0027 (9)	0.0036 (8)	0.0003 (8)
C4	0.0529 (11)	0.0519 (10)	0.0424 (10)	0.0016 (9)	0.0124 (8)	0.0016 (8)
C5	0.0512 (11)	0.0659 (12)	0.0529 (11)	−0.0090 (9)	0.0073 (9)	−0.0066 (10)
C6	0.0510 (11)	0.0671 (12)	0.0412 (10)	−0.0063 (9)	0.0031 (8)	−0.0078 (9)
C7	0.0556 (11)	0.0570 (11)	0.0373 (9)	0.0036 (9)	0.0064 (8)	−0.0005 (8)
C8	0.0548 (11)	0.0487 (10)	0.0379 (9)	0.0054 (8)	0.0088 (8)	0.0017 (8)
C9	0.0526 (11)	0.0641 (12)	0.0551 (12)	−0.0006 (10)	0.0069 (9)	−0.0011 (10)
C10	0.0585 (12)	0.0690 (13)	0.0607 (13)	−0.0011 (10)	0.0186 (10)	−0.0012 (11)
C11	0.0678 (14)	0.0945 (16)	0.0401 (11)	−0.0064 (12)	0.0029 (10)	−0.0083 (10)

### Geometric parameters (Å, °)

**Table:**

S1—C10	1.691 (2)	C2—C3	1.383 (2)
S1—C8	1.7239 (19)	C3—C4	1.357 (3)
N1—O1	1.225 (2)	C3—H3A	0.9300
N1—O2	1.225 (2)	C4—C5	1.395 (3)
N1—C2	1.447 (2)	C5—C6	1.359 (3)
N2—O4	1.219 (2)	C5—H5	0.9300
N2—O3	1.221 (2)	C6—H6	0.9300
N2—C4	1.454 (2)	C7—C8	1.454 (3)
N3—C1	1.356 (2)	C7—C11	1.505 (3)
N3—N4	1.373 (2)	C9—C10	1.352 (3)
N3—H3	0.8600	C9—H9	0.9300
N4—C7	1.290 (2)	C10—H10	0.9300
N5—C8	1.324 (2)	C11—H11A	0.9600
N5—C9	1.355 (3)	C11—H11B	0.9600
C1—C6	1.405 (2)	C11—H11C	0.9600
C1—C2	1.420 (2)		
			
C10—S1—C8	89.63 (10)	C6—C5—C4	119.59 (18)
O1—N1—O2	122.43 (17)	C6—C5—H5	120.2
O1—N1—C2	118.64 (15)	C4—C5—H5	120.2
O2—N1—C2	118.92 (17)	C5—C6—C1	122.14 (17)
O4—N2—O3	123.44 (18)	C5—C6—H6	118.9
O4—N2—C4	118.47 (18)	C1—C6—H6	118.9
O3—N2—C4	118.07 (18)	N4—C7—C8	126.66 (16)
C1—N3—N4	116.93 (15)	N4—C7—C11	114.95 (17)
C1—N3—H3	121.5	C8—C7—C11	118.38 (17)
N4—N3—H3	121.5	N5—C8—C7	124.57 (17)
C7—N4—N3	118.86 (16)	N5—C8—S1	113.67 (14)
C8—N5—C9	110.35 (17)	C7—C8—S1	121.76 (13)
N3—C1—C6	120.08 (16)	C10—C9—N5	115.95 (19)
N3—C1—C2	123.59 (16)	C10—C9—H9	122.0
C6—C1—C2	116.32 (16)	N5—C9—H9	122.0
C3—C2—C1	121.21 (16)	C9—C10—S1	110.40 (16)
C3—C2—N1	116.32 (15)	C9—C10—H10	124.8
C1—C2—N1	122.46 (16)	S1—C10—H10	124.8
C4—C3—C2	119.92 (17)	C7—C11—H11A	109.5
C4—C3—H3A	120.0	C7—C11—H11B	109.5
C2—C3—H3A	120.0	H11A—C11—H11B	109.5
C3—C4—C5	120.79 (18)	C7—C11—H11C	109.5
C3—C4—N2	119.92 (17)	H11A—C11—H11C	109.5
C5—C4—N2	119.30 (18)	H11B—C11—H11C	109.5

### Hydrogen-bond geometry (Å, °)

**Table:**

*D*—H···*A*	*D*—H	H···*A*	*D*···*A*	*D*—H···*A*
N3—H3···O1	0.86	2.03	2.628 (2)	126
N3—H3···N5	0.86	2.03	2.686 (2)	133
C9—H9···O1^i^	0.93	2.58	3.289 (3)	133

Symmetry codes: (i) −*x*, −*y*, −*z*+1.
